# Suicide and Ambient Temperature in East Asian Countries: A Time-Stratified Case-Crossover Analysis

**DOI:** 10.1289/ehp.1409392

**Published:** 2015-06-12

**Authors:** Yoonhee Kim, Ho Kim, Yasushi Honda, Yue Leon Guo, Bing-Yu Chen, Jong-Min Woo, Kristie L. Ebi

**Affiliations:** 1 Department of Pediatric Infectious Diseases, Institute of Tropical Medicine, Nagasaki University, Nagasaki, Japan; 2 Graduate School of Public Health, Seoul National University, Seoul, South Korea; 3 Faculty of Health and Sport Sciences, University of Tsukuba, Tsukuba, Ibaraki, Japan; 4 Environmental and Occupational Medicine, National Taiwan University College of Medicine and National Taiwan University Hospital, Taipei, Taiwan (Republic of China); 5 Department of Psychiatry, Seoul Paik Hospital, Inje University School of Medicine, Seoul, South Korea; 6 Stress Research Institute, Inje University, Seoul, South Korea; 7 University of Washington, Seattle, Washington, USA

## Abstract

**Background:**

A limited number of studies suggest that ambient temperature contributes to suicide; these studies typically focus on a single nation and use temporally and spatially aggregated data.

**Objective:**

We evaluated the association between ambient temperature and suicide in multiple cities in three East Asian countries.

**Methods:**

A time-stratified case-crossover method was used to explore the relationship between temperature and suicide, adjusting for potential time-varying confounders and time-invariant individual characteristics. Sex- and age-specific associations of temperature with suicide were estimated, as were interactions between temperature and these variables. A random-effects meta-analysis was used to estimate country-specific pooled associations of temperature with suicide.

**Results:**

An increase in temperature corresponding to half of the city-specific standard deviation was positively associated with suicide in most cities, although average suicide rates varied substantially. Pooled country-level effect estimates were 7.8% (95% CI: 5.0, 10.8%) for a 2.3°C increase in ambient temperature in Taiwan, 6.8% (95% CI: 5.4, 8.2%) for a 4.7°C increase in Korea, and 4.5% (95% CI: 3.3, 5.7%) for a 4.2°C increase in Japan. The association between temperature and suicide was significant even after adjusting for sunshine duration; the association between sunshine and suicide was not significant. The associations were greater among men than women in 12 of the 15 cities although not significantly so. There was little evidence of a consistent pattern of associations with age. In general, associations were strongest with temperature on the same day or the previous day, with little evidence of associations with temperature over longer lags (up to 5 days).

**Conclusions:**

We estimated consistent positive associations between suicide and elevated ambient temperature in three East Asian countries, regardless of country, sex, and age.

**Citation:**

Kim Y, Kim H, Honda Y, Guo YL, Chen BY, Woo JM, Ebi KL. 2016. Suicide and ambient temperature in East Asian countries: a time-stratified case-crossover analysis. Environ Health Perspect 124:75–80; http://dx.doi.org/10.1289/ehp.1409392

## Introduction

Suicide is a crucial public health concern. In East Asia (a sub-region of Asia), suicide rates in South Korea, Japan, and Taiwan have increased to high levels. Suicide rates in South Korea had a notable spike from 13.6 per 100,000 in 1997 to 18.8 per 100,000 in 1998 in the wake of the economic crisis, and the rate rose even higher in 2010 (33.5 per 100,000). Japan experienced a sudden rise in suicide during the economic crisis from 15.2 per 100,000 in 1997 to 20.4 per 100,000 in 1998; this rate persists to this day (21.2 per 100,000 in 2010) [Organisation for Economic Co-operation and Development (OECD) 2011, 2012]. The suicide rate in Taiwan increased from 10.0 per 100,000 in 1998 to 19.3 per 100,000 in 2006 and then decreased to 16.8 per 100,000 in 2010 (Lee and Liao 2012). In comparison, suicide rates in OECD countries average 11.3 per 100,000 in 2009 (OECD 2011).

Suicide is affected by various factors and by complex interactions between society and individuals (Chang et al. 2009; Durkheim 1951; Juurlink et al. 2004; Qin et al. 2003). However, there is a consistent seasonal pattern (Ajdacic-Gross et al. 2010). A century ago, suicides peaked in spring or early summer in many European countries (Durkheim 1951). Since then, many studies have suggested seasonal variation of suicide rates with a peak in spring or early summer, stronger in men than in women, stronger in the elderly than in young people, and stronger in suicide by violent methods (i.e., hanging, drowning, jumping, or cutting) compared with nonviolent methods (i.e., ingestion of poisons, drugs, gases, or vapors) (Christodoulou et al. 2012; Lin et al. 2008; Reutfors et al. 2009). Interestingly, these patterns have been observed across geographic regions, including European countries (Switzerland, Italy, Finland, and Romania), the United States, countries located in the Southern hemisphere (Australia and South Africa; showing reciprocal patterns with the Northern hemisphere), and Asian countries (Japan, Hong Kong, and China) that have different climates, economic status, and cultures (Ajdacic-Gross et al. 2010; Christodoulou et al. 2012; Sun et al. 2011).

Studies designed to explain this pattern provided contradictory evidence of an association between ambient temperature and suicide, at least in part because the methods used had limited temporal and spatial variations or used aggregated data (Deisenhammer 2003; Dixon et al. 2007; Dixon and Kalkstein 2009; Ruuhela et al. 2009; Tsai 2010; Woo et al. 2012). Although a few studies endeavored to apply more appropriate statistical models that considered temporal variations and adjusted for seasonal trends using, for example, time-series regression models (Dixon et al. 2014; Kim et al. 2011; Lee et al. 2006; Likhvar et al. 2011; Lin et al. 2008; Page et al. 2007). Most of these studies covered a single nation, and few took individual characteristics (sex or age) into account to compare associations of temperature with suicide between groups; those that did so generally used stratification approaches (Kim et al. 2011; Page et al. 2007).

To improve upon these previous studies, we used a time-stratified case-crossover approach to adjust for time-invariant individual characteristics. This standardized method was applied across multiple locations in three East Asian countries to examine the relationship between ambient temperature and suicide and to evaluate sex and age differences.

## Methods

**Figure 1:**
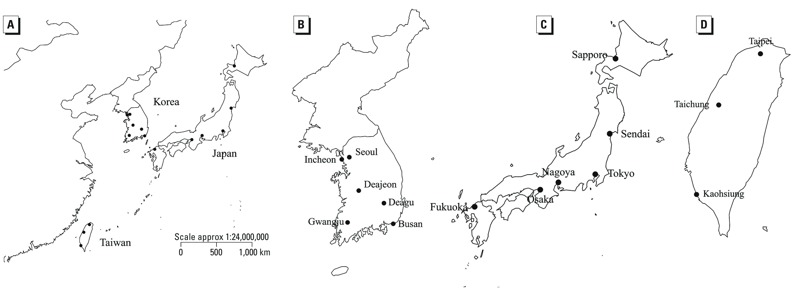
Geographical locations of the study area (*A*), six cities in South Korea (*B*), six cities in Japan (*C*), and three cities in Taiwan (*D*).

*Study area.* We selected 15 widely distributed major cities (with populations of over 1 million in 2010) from three countries: South Korea (6 cities), Japan (6 cities), and Taiwan (3 cities) (Figure 1; see also Supplemental Material, Table S1). The study period varied across countries based on access to mortality data: 19 years in Korea (1992–2010), 39 years in Japan (1972–2010), and 14 years in Taiwan (1994–2007). The neighboring countries, Korea and Japan, are distinctly seasonal; that is, temperature and relative humidity are high in summer, and winters are cold and dry. The climate of Japan, in contrast with that of Korea, varies between the northernmost and southernmost chain of islands; the difference in mean temperature between Sapporo (the northernmost city) and Fukuoka (the southernmost city) was 8.0°C. The climate of Taiwan is subtropical with a mild winter and year-round high humidity.

*Suicide data.* In all 15 cities, deaths were considered to be suicide if the death was coded as intentional self-poisoning and self-harm from the *International Classification of Diseases, 8th–10th Revisions, Clinical Modification* (ICD-8, ICD-9, and ICD-10). The ICD-8 and ICD-9 codes used were E950.0–E958.9, and the ICD-10 codes used were X60–X84. Suicide data were obtained from Statistics Korea, Ministry of Strategy and Finance in South Korea; the Ministry of Health, Labour and Welfare in Japan; and the Department of Statistics, Ministry of Health and Welfare in Taiwan (unpublished data, available by special permission from the agencies). Suicide cases were categorized by sex and by age groups, excluding cases < 10 years of age. Age was categorized into three groups: 10–24 years (adolescents and young adults), 25–64 years, and ≥ 65 years (older adults). Additionally, we collected suicide data at the national level to compare suicide rates across time periods between countries. City-specific population data were used to calculate yearly suicide rates in each city: 5-year census with linear interpolation for Korea and Japan, and single-year for Taiwan. The population data were obtained from Statistics Korea, Ministry of Strategy and Finance in South Korea (http://kosis.kr/); the Statistics Bureau, Ministry of Internal Affairs and Communications in Japan (http://www.e-stat.go.jp/SG1/estat/eStatTopPortal.do); and the Department of Statistics, Ministry of the Interior in Taiwan (http://www.moi.gov.tw/stat/).

*Weather data.* Hourly weather variables monitored at meteorological observatories in each city were obtained from the Korea Meteorological Administration, the Japan Meteorological Agency, and the Taiwan Central Weather Bureau. We calculated daily mean ambient temperature (degrees Celsius), relative humidity (percent), atmospheric pressure (hectopascals), and the daily sum of sunshine duration (hours) at the city level.

*Statistical analyses.* Two-stage analyses were conducted to examine the association between ambient temperature and suicide. In the first stage, we conducted city-specific analyses using a time-stratified case-crossover design that adjusted for seasonal patterns and long-term trends in the design itself (Barnett and Dobson 2010). Since the development of the case-crossover design, the time-stratified approach has been recognized as being the least-biased method among referent (control) selection strategies (Basu et al. 2005; Janes et al. 2005). *A priori,* we split the time series into 56-day nonoverlapping strata and compared differences in exposure (temperature) between case and control days within the same stratum using a conditional logistic regression model. Each case was matched to 7 control days on the same day of the week during the 56-day stratum. Because each case served as its own control, case and control days were automatically matched with regard to age and sex. We used a 56-day (8-week) time period instead of a 28-day (4-week) time period to ensure that there was a least one suicide during each time period in the smaller cities. In the statistical models, the association between temperature and suicide was estimated adjusting for sunshine duration, relative humidity, atmospheric pressure, time trend (date of suicide; as a linear term), and month. A possible bias in temperature–suicide associations attributable to the longer stratum (Guo and Barnett 2015) was reduced by adjusting for the time trend and month in the models. In our sensitivity analyses, the difference in the risk estimates between the 28- and 56-day strata was smaller in the model that adjusted for month and date than the difference in those in the model that did not adjust for the two variables.

We specified two sets of models: one to estimate overall associations and the second with interaction terms to estimate sex- and age-specific associations. The case-crossover design canceled out potential confounding by time-invariant variables such as sex and age; thus, there was a need to adjust for these characteristics. By using interactions, we could estimate the sex- and age-specific associations between temperature and suicide and test statistical significances for temperature-related sex and age differences. We modeled interactions between temperature and sex or categorical age group (10–24 years and 25–64 years, with ≥ 65 years as the reference category) using separate models.

Because the ranges of ambient temperature varied across the three countries, we calculated the associations of temperature with suicide corresponding to an SD/2-unit (standard deviation of the mean temperature divided by 2) increase of each city’s mean temperature to show comparable results. In particular, the range of mean temperatures between winter and summer in the cities in Taiwan was narrower (from 17.2°C to 28.9°C) than those in the cities in Korea (from –0.1°C to 25.2°C) and Japan (from –2.7°C to 26.5°C); thus, the units of temperature (SD/2) in the Taiwanese cities were smaller (from 1.9°C to 2.6°C) than those in the Korean (from 4.0°C to 5.1°C) and Japanese (from 3.9°C to 4.8°C) cities.

In the second stage, we performed a meta-analysis with random effects to produce pooled associations. Because there was heterogeneity between city-specific associations in 15 cities (*I*^2^ = 40.8%, and *p* = 0.02 derived from a chi-square test on Cochran’s *Q* statistic) (Higgins and Thompson 2002), the meta-analysis was conducted separately in each country. Country-specific averages of the units of temperature (SD/2) were used to show the results. Similarly, the sex- and age-specific associations were pooled by country.

*Sensitivity analyses.* We performed several sensitivity analyses. First, we compared the association between temperature and suicide adjusting for month and date with the unadjusted association for temperature based on 28- and 56-day strata in national capital cities (Seoul, Tokyo, and Taipei) to examine whether a 56-day stratum was appropriate. Second, we compared the association between temperature and suicide based on models with (main model) and without adjusting for sunshine duration. Simultaneously, we estimated associations between sunshine duration and suicide with and without temperature in the models. To examine the lag effects of temperature and sunshine on suicide, we specified two types of lag structures: single and moving averages from event day to 5 days before. In addition, moving averages of sunshine duration were extended to 10, 15, 20 days, and a 7-day moving average 90 days before the suicide was used on the assumption that a depressive mood in winter related to seasonal affective disorder (SAD) might have influenced spring suicides (Praschak-Rieder et al. 2008; Preti 1997).

We used SAS version 9.3 (SAS Institute Inc., Cary, NC, USA) for the city-specific analyses and the “metafor” package of R version 3.0.1 (R Core Team 2014) for the meta-analyses. The alpha level used to define statistical significance was 0.05.

## Results

**Figure 2:**
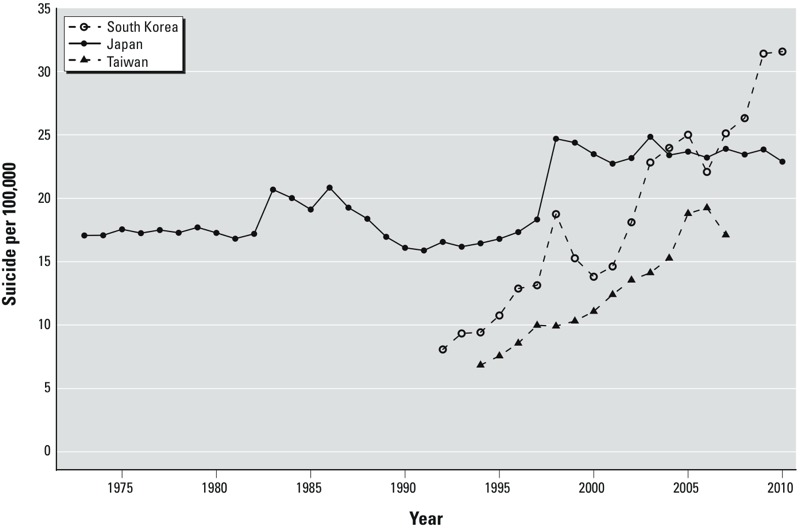
Yearly trend of suicide rates at the national level. Suicide rates differed significantly over time: a sharp increase occurred in South Korea, a sudden rise has taken place since 1998 in Japan, and a decline began in 2007 after a steady increase in Taiwan. Suicide and population data were obtained from Statistics Korea, Ministry of Strategy and Finance in South Korea; the Ministry of Health, Labour and Welfare (for suicide) and Statistics Bureau, Ministry of Internal Affairs and Communications (for population) in Japan; and the Department of Statistics, Ministry of Health and Welfare (for suicide) and Department of Statistics, Ministry of the Interior (for population) in Taiwan.

Figure 2 shows yearly trends in suicide rates (national levels) across South Korea, Japan, and Taiwan. The suicide rates differed significantly over time and confirmed reported patterns: a sharp increase in South Korea, a rise since 1998 in Japan, and a decline in 2007 after a steady increase in Taiwan. Suicide rates also differed by sex and age across the three countries (see Supplemental Material, Figures S1 and S2). For example, suicides by elderly adults in South Korea increased 392% between 1992 and 2010, whereas those in Japan decreased consistently. In addition, male and female suicides increased in South Korea and Taiwan, whereas female suicides in Japan remained stable. The seasonal peak of suicide occurred in spring and early summer (March–June) but extended until July in two Taiwanese cities (Taipei and Kaohsiung) (see Supplemental Material, Figure S3). There was also a consistent pattern of suicides on certain days of the week across the cities—more suicides on Mondays and Tuesdays and fewer suicides on Saturdays and Sundays (see Supplemental Material, Figure S4). Climate features varied between the study cities by location—annual average temperature ranged from 8.8°C (Sapporo) to 25.3°C (Kaohsiung) (Table 1). Relative humidity was similar among the cities in Korea and Japan, humid in summer but dry in winter, whereas the Taiwanese cities, which possessed a sub-tropical climate, were humid all year round (see Supplemental Material, Table S2).

**Table 1:** Suicide rate, daily average of total suicides, and weather activity for 15 cities.

Country/city	Study period	Total suicide cases	Suicide rate (per 100,000)	Daily suicide cases (mean ± SD)	Temperature (°C) (mean ± SD)	Sunshine (hours) (mean ± SD)	Humidity (%) (mean ± SD)	Atmospheric pressure (hPa) (mean ± SD)
Korea
Seoul	1992–2010	28,134	14.9	4.1 ± 2.8	12.8 ± 10.1	5.4 ± 3.7	62.9 ± 14.6	1,016.2 ± 8.1
Busan	1992–2010	12,922	19.0	1.9 ± 1.6	14.9 ± 8.0	6.2 ± 3.9	63.7 ± 18.5	1,015.5 ± 7.2
Incheon	1992–2010	8,889	18.8	1.3 ± 1.3	12.5 ± 9.7	6.2 ± 3.9	67.9 ± 14.3	1,015.9 ± 8.1
Daegu	1992–2010	7,631	16.3	1.1 ± 1.2	14.4 ± 9.3	6.1 ± 3.8	59.4 ± 15.5	1,016.4 ± 7.7
Daejeon	1992–2010	4,622	17.3	0.7 ± 0.9	13.0 ± 9.8	5.9 ± 3.7	67.1 ± 13.5	1,016.3 ± 8.0
Gwangju	1992–2010	3,826	14.5	0.6 ± 0.8	14.1 ± 9.3	5.7 ± 3.7	67.6 ± 12.7	1,016.2 ± 7.9
Japan
Sapporo	1972–2010	11,598	18.0	0.8 ± 1.0	8.8 ± 9.5	5.6 ± 3.7	69.7 ± 10.8	1,012.5 ± 7.1
Sendai	1972–2010	5,671	16.6	0.4 ± 0.7	12.3 ± 8.2	6.1 ± 3.5	71.1 ± 13.4	1,014.1 ± 6.8
Tokyo	1972–2010	60,184	18.4	4.2 ± 2.3	16.2 ± 7.8	6.4 ± 3.5	62.2 ± 15.4	1,013.8 ± 6.8
Nagoya	1972–2010	15,231	18.2	1.1 ± 1.1	15.7 ± 8.4	6.7 ± 3.5	66.7 ± 12.7	1,014.7 ± 6.5
Osaka	1972–2010	24,955	24.1	1.8 ± 1.4	16.8 ± 8.3	6.3 ± 3.5	63.6 ± 10.9	1,015.0 ± 6.7
Fukuoka	1972–2010	9,066	18.7	0.6 ± 0.8	16.8 ± 7.8	6.1 ± 3.7	68.1 ± 11.6	1,015.4 ± 7.0
Taiwan
Taipei	1994–2007	9,480	10.8	1.9 ± 1.6	23.2 ± 5.3	4.0 ± 3.7	76.1 ± 9.1	1,012.5 ± 6.8
Taichung	1994–2007	3,350	9.7	0.7 ± 0.8	23.7 ± 4.7	5.8 ± 3.5	74.8 ± 7.7	1,002.6 ± 5.6
Kaohsiung	1994–2007	5,049	13.2	1.0 ± 1.1	25.3 ± 3.8	6.1 ± 3.4	76.0 ± 7.1	1,012.0 ± 5.4

**Figure 3:**
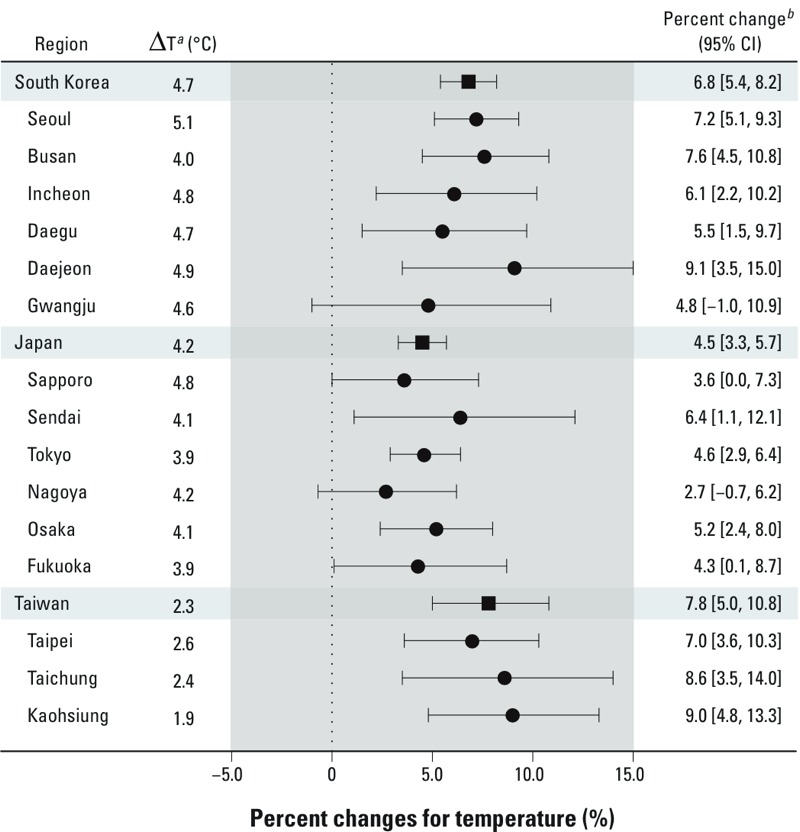
Country- (squares) and city- (circles) specific associations of temperature on the same day with suicide. ***^a^***∆T indicates an SD/2-unit increase of each city’s mean temperature (standard deviation of mean temperature divided by 2). ***^b^***Percent change (%) indicates suicide risk corresponding to an SD/2 increase in mean temperature adjusting for sunshine duration, relative humidity, atmospheric pressure, time trend (date of suicide), and month. The country-specific pooled associations (squares) were calculated by a random-effect meta-analysis.

*Associations between temperature and suicide.* Figure 3 presents country- and city-specific associations between temperature and suicide, showing that higher ambient temperature was associated with a significant increase in suicide in 12 of the 15 cities. The highest country-specific pooled associations for temperature were 7.8% (95% CI: 5.0, 10.8%) for a 2.3°C increase in ambient temperature in Taiwan; 6.8% (95% CI: 5.4, 8.2%) for a 4.7°C increase in Korea; and 4.5% (95% CI: 3.3, 5.7%) for a 4.2°C increase in Japan. The range of city-specific associations with temperature was broader in Korea (4.8–9.1%, 6 cities) and Japan (2.7–6.4%, 6 cities) than in Taiwan (7.0–9.0%, 3 cities). Associations based on models with and without adjustment for sunshine duration were comparable (see Supplemental Material, Table S3). There was a significant positive association between sunshine duration and suicide in 2 cities before adjustment for temperature (see Supplemental Material, Table S3), but there were no significant associations after adjustment for temperature, including sunshine duration during single-day lag periods up to 5 days (see Supplemental Material, Figure S5) and based on models with moving averages up to 90 days (see Supplemental Material, Figure S6).

**Figure 4:**
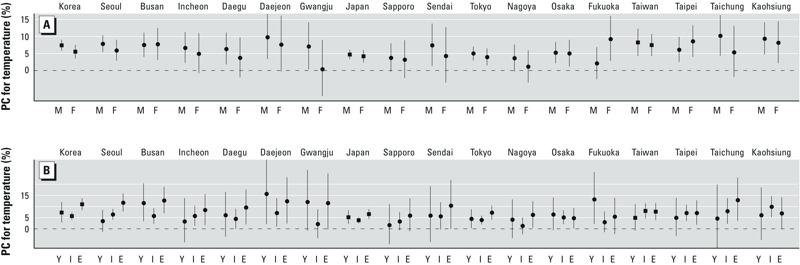
Associations between temperature and suicide by sex (*A*) and by age group (*B*). City- (circles) and country- (squares) specific associations of temperature with suicide are indicated by percent change (PC) of suicide risk corresponding to an SD/2 increase (standard deviation divided by 2) in mean temperature. Abbreviations: E, ≥ 65 years of age; F, female; I, 25–64 years of age; M, male; Y, 10–24 years of age.

*Subgroup analyses.* Figure 4 and Supplemental Material, Table S4, show sex- and age-specific associations between temperature and suicide by city. The association was stronger among men than among women in 12 of 15 cities (although not significantly so), with the exception of Busan, Taipei, and Fukuoka (the last being the only city where there was a significant difference by sex). In contrast, there was little evidence of association with age. In 9 of 15 cities, the association was stronger in those ≥ 65 years of age than in the other age groups, but it was statistically significant in 3 cities (Seoul, Busan, and Tokyo). The association in Seoul was significantly stronger among those ≥ 65 years of age than in those 10–24 or 25–64 years of age, but in Busan and Tokyo, the association was significantly weaker among those 25–64 years of age than in those ≥ 65 years of age.

*Lag structure.* Compared with longer single-day lag periods (up to 5 days), the association of temperature with suicide was strongest for temperature on the same day (lag = 0) in most cities, with the exceptions of Busan, Nagoya, and Fukuoka, where the association was slightly stronger with temperature on the previous day (lag = 1) (see Supplemental Material, Figure S7). The associations were 7.8% (95% CI: 4.8, 10.9%) for a 4.0°C increase in Busan, 4.2% (95% CI: 0.9, 7.7%) for a 4.2°C increase in Nagoya, and 5.9% (95% CI: 1.6, 10.4%) for a 3.9°C increase in Fukuoka. In general, the association decreased as the number of lag days increased. Associations with moving average temperatures up to 5 days showed less variation than associations based on single-day lags (see Supplemental Material, Figure S8).

## Discussion

The aim of this study was to examine the relationship between suicide and ambient temperature across multiple sites with large discrepancies in suicide rates. Higher temperatures were associated with significantly higher risks of suicide in most cities. In addition, the association between temperature and suicide did not differ significantly by sex or age. These findings suggest that in East Asia, suicide is positively associated with ambient temperature, regardless of country, sex, or age.

Previous studies reported some evidence supporting a positive association between high ambient temperature and suicide (Christodoulou et al. 2012; Deisenhammer 2003; Deisenhammer et al. 2003; Kim et al. 2011; Likhvar et al. 2011; Page et al. 2007). For example, two studies in East Asia using a time series analysis found a 1.4% increase in suicide associated with each 1°C increase in daily mean temperature in South Korea and increases in suicide associated with temperature on the same day (lag = 0) for all regions in Japan (Kim et al. 2011; Likhvar et al. 2011). In addition, a study in Austria that used hierarchical logistic regression reported a 12% increase in suicide risk associated with a 10°C increase in temperature (Deisenhammer et al. 2003). A study in England and Wales that used a threshold model showed that each 1°C increase in mean temperature above 18°C was associated with a 3.8% increase in suicide (Page et al. 2007).

*Possible mechanisms for temperature to trigger suicide.* There are no agreed-upon biological mechanisms to explain the association between ambient temperature and suicide. It has been hypothesized that higher suicide rates in the spring and early summer may reflect seasonal variations in serotonin, a neurotransmitter that may influence impulsiveness and aggression, possibly leading to suicide (Luykx et al. 2013; Mann 2013).

L-Tryptophan is a serotonin precursor, and low concentrations of L-tryptophan in plasma are associated with major depression disorders (Ogawa et al. 2014). An ecological study in Belgium (Maes et al. 1995) showed that seasonal variations in the concentration of plasma L-tryptophan (in blood samples collected monthly from healthy volunteers) were inversely correlated with the seasonal variation of the number of violent suicides. In addition, the authors reported that these concentrations were negatively associated with ambient temperature.

Decreased levels of the serotonin transporter in the midbrain or prefrontal cortex (PFC) have been suggested to be associated with depressed suicides (Mann 2013; Miller et al. 2013). Praschak-Rieder et al. (2008) reported that serotonin transporter bindings, when measured at several brain regions in healthy subjects, were lower in spring and summer than in fall and winter and were negatively correlated with daily sunshine duration. These findings suggest a possible relationship between seasonal variation of serotonin transporters and suicide.

Because sunshine has been reported to be associated with alterations in the serotonergic system (Lambert et al. 2002; Praschak-Rieder et al. 2008; Sansone and Sansone 2013) and with suicide rate in Australia and in Austria (Lambert et al. 2003; Vyssoki et al. 2014), we analyzed whether sunshine duration was associated with suicide. We found no evidence of an association between sunshine duration and suicide in our study cities, regardless of the lag period.

*Subgroups.* The literature suggests that the association between temperature and suicide is generally stronger in men and older adults. A population-based ecological study in South Korea (Kim et al. 2011) reported a higher risk of suicide with elevated ambient temperature among men than among women, as well as a higher rate among those ≥ 65 years of age than among those < 65 years of age. Another population-based study in Italy using monthly data (Preti and Miotto 1998) reported that seasonality of suicide was more prevalent among older men and women (≥ 65 years) than among younger people (14–24 or 25–64 years) and that the simple correlation between violent suicide and temperature was higher in men than in women. The patterns for sex that we found in our study were similar to those of previous studies, with the association between suicide and temperature generally stronger in men than in women (in 12 of 15 cities); however, sex was not significantly associated with suicide. In addition, age differences were not statistically significant except in three large cities (stronger association in those ≥ 65 years of age than in all other age groups in Seoul, and stronger associations in those ≥ 65 years of age than in those 25–64 years of age in Busan and Tokyo). The age difference for large cities most likely resulted from the larger sample sizes.

*Strengths.* To our knowledge, this is the first study of suicide and temperature using a time-stratified case-crossover analysis that covered multiple locations in multiple countries, adjusted for seasonal variations, and accounted for time-invariant individual characteristics. Moreover, our study covered long-term periods ranging from 14 years (Taiwan) to 39 years (Japan). Most previous studies examining the association between suicide and ambient temperature were conducted in a single country using aggregated data owing to a small number of suicide cases, although a few used more advanced statistical methods (Kim et al. 2011; Lee et al. 2006; Likhvar et al. 2011; Lin et al. 2008; Page et al. 2007). In this study, we tested the statistical significance of the associations between suicide and temperature by sex and by age group, incorporating interaction terms into the models. Previous studies using time-series analysis utilized the stratification method to compare associations by sex or age (Kim et al. 2011; Page et al. 2007). The model with interactions that we used in our study allowed the statistical comparison of associations by sex or age, yielding parameter estimates of the difference based on the whole dataset.

*Limitations.* This study had some limitations. First, generalization of our results might be limited because the study area only comprised countries in East Asia. Although we found consistent associations between high ambient temperatures and suicide regardless of country, additional studies that include other countries, in particular countries with different suicide rates, are needed to confirm our results. Second, our findings were estimated on the basis of a linear association between suicide and temperature. Although higher temperature was positively associated with suicide, there were regional differences in whether suicide risks increased during extreme high-temperature periods (Dixon et al. 2014; Kim et al. 2011; Likhvar et al. 2011; Page et al. 2007). Additional studies with a wider range of regions are needed to assess suicide risks associated with extremely high temperatures. Third, we could not consider air pollution in our analyses. Studies have recently reported an association between air pollution (particulate matter) and suicide or depression (Kim et al. 2010; Lim et al. 2012). Evaluating the interactions between ambient temperature, air pollution, and suicide presents additional challenges. Finally, it was not possible to consider depression in our analyses because of difficulties in collecting the data. Depression is a risk factor for suicide (Qin et al. 2003) and is associated with season, for example, seasonal affective disorder (Magnusson 2000). Assessing the association between mental illness and temperature would provide increased understanding of the impact of temperature on suicide.

## Conclusions

Our study suggests that elevated ambient temperature may increase suicidal behaviors. This study provides an important consideration for suicide prevention. With respect to climate variability and change, research and policy interests have focused on heat-related deaths due to physical illnesses, but our findings support the need for research on the potential effects of a changing climate on mental illnesses as well.

## Supplemental Material

(701 KB) PDF
