# Supplementary material for: Suicide and Ambient Temperature in East Asian Countries: A Time-Stratified Case-Crossover Analysis
Source: Environ Health Perspect. 2015 Jun 12;124(1):75–80. doi: 10.1289/ehp.1409392 (PMC4710603; doi:10.1289/ehp.1409392)
Supplement: (701 KB) PDF [file ehp.1409392.s001.acco.pdf]

Environ Health Perspect
DOI: 10.1289/ehp.1409392

Note to Readers: *EHP* strives to ensure that all journal content is accessible to all readers. However, some figures and Supplemental Material published in *EHP* articles may not conform to 508 standards due to the complexity of the information being presented. If you need assistance accessing journal content, please contact ehp508@niehs.nih.gov. Our staff will work with you to assess and meet your accessibility needs within 3 working days.

Supplemental Material

Suicide and Ambient Temperature in East Asian Countries: A Time-Stratified Case-Crossover Analysis

Yoonhee Kim, Ho Kim, Yasushi Honda, Yue Leon Guo, Bing-Yu Chen, Jong-Min Woo, and Kristie L. Ebi

Table of Contents

Table S1. City-specific characteristics of suicide cases and population size.

Table S2. Weather characteristics by season in fifteen cities.

Table S3. The associations for temperature or sunshine duration.

Table S4. City-specific associations between same-day temperature and suicide according to gender and age group.

Figure S1. Yearly trend of suicide rates on national levels by gender in Korea, Japan, and Taiwan. Suicide and population data were obtained from Statistics Korea, Ministry of Strategy and Finance in South Korea, Ministry of Health, Labor and Welfare (for suicide) and Statistics Bureau, Ministry of Internal Affairs and Communications (for population) in Japan, and Department of Statistics, Ministry of Health and Welfare (for suicide) and Department of Statistics, Ministry of the Interior (for population) in Taiwan.

Figure S2. Yearly trend of suicide rates on national levels by age groups in Korea, Japan, and Taiwan. Suicide and population data were obtained from Statistics Korea, Ministry of Strategy and Finance in South Korea, Ministry of Health, Labor and Welfare (for suicide) and Statistics Bureau, Ministry of Internal Affairs and Communications (for population) in Japan, and

Department of Statistics, Ministry of Health and Welfare (for suicide) and Department of Statistics, Ministry of the Interior (for population) in Taiwan.

Figure S3. Monthly trend of suicide in fifteen cities. Study period varied depending on the country: Korea (1992–2010), Japan (1972–2010), and Taiwan (1994–2007). Suicide data were obtained from Statistics Korea, Ministry of Strategy and Finance in South Korea, Ministry of Health, Labor and Welfare in Japan, and Department of Statistics, Ministry of Health and Welfare in Taiwan.

Figure S4. Trends for day of the week of suicide in fifteen cities. Study period varied depending on the country: Korea (1992–2010), Japan (1972–2010), and Taiwan (1994–2007). Suicide data were obtained from Statistics Korea, Ministry of Strategy and Finance in South Korea, Ministry of Health, Labor and Welfare in Japan, and Department of Statistics, Ministry of Health and Welfare in Taiwan.

Figure S5. Lagged effect estimates for sunshine on suicide according to single lags. PC indicates percent change of suicide risk corresponding to a SD/2-increase of mean sunshine, adjusting for temperature, relative humidity, atmospheric pressure, long-term time-trend, and month.

Figure S6. Lagged effect estimates for sunshine on suicide according to moving averages. Moving average 90 indicates the average of sunshine for a week on previous ninety days. PC indicates percent change of suicide risk corresponding to a SD/2-increase of mean sunshine, adjusting for temperature, relative humidity, atmospheric pressure, long-term time-trend, and month.

Figure S7. Lagged effect estimates for temperature on suicide according to single lags. PC indicates percent change of suicide risk corresponding to a SD/2-increase of mean temperature, adjusting for sunshine duration, relative humidity, atmospheric pressure, long-term time-trend, and month.

Figure S8. Lagged effect estimates for temperature on suicide according to moving averages. PC indicates percent change of suicide risk corresponding to a SD/2-increase of mean temperature, adjusting for sunshine duration, relative humidity, atmospheric pressure, long-term time-trend, and month.

Table S1. City-specific characteristics of suicide cases and population size.

Country	City	Study period	Population size[a]	Total suicide cases	Yearly suicide cases (mean ± SD)	Monthly suicide cases (mean ± SD)	Daily suicide cases (mean ± SD)	Suicide rate (per 100,000)
Korea	Seoul	1992-2010	9 935 227	28 134	1480.7 ± 651.0	123.4 ± 13.8	4.1 ± 2.8	14.9
	Busan	1992-2010	3 603 935	12 922	680.1 ± 291.6	56.7 ± 7.0	1.9 ± 1.6	19.0
	Inchoen	1992-2010	2 494 279	8 889	467.8 ± 214.9	39 ± 4.9	1.3 ± 1.3	18.8
	Daegu	1992-2010	2 460 241	7 631	401.6 ± 198.5	33.5 ± 3.0	1.1 ± 1.2	16.3
	Daejeon	1992-2010	1 396 261	4 622	243.3 ± 117.0	20.3 ± 2.7	0.7 ± 0.9	17.3
	Gwangju	1992-2010	1 375 974	3 826	201.4 ± 112.5	16.8 ± 2.4	0.6 ± 0.8	14.5
Japan	Sapporo	1972-2010	1 653 862	11 598	297.4 ± 103.9	24.8 ± 2.5	0.8 ± 1.0	18.0
	Sendai	1972-2010	868 688	5 671	145.4 ± 59.0	12.1 ± 0.9	0.4 ± 0.7	16.6
	Tokyo	1972-2010	8 381 781	60 184	1543.2 ± 286.7	128.6 ± 8.0	4.2 ± 2.3	18.4
	Nagoya	1972-2010	2 155 189	15 231	390.5 ± 63.3	32.5 ± 2.2	1.1 ± 1.1	18.2
	Osaka	1972-2010	2 647 817	24 955	639.9 ± 151.5	53.3 ± 3.5	1.8 ± 1.4	24.1
	Fukuoka	1972-2010	1 247 447	9 066	232.5 ± 71.0	19.4 ± 1.3	0.6 ± 0.8	18.7
Taiwan	Taipei	1994-2007	6 188 566	9 480	677.1 ± 296.2	56.4 ± 3.8	1.9 ± 1.6	10.8
	Taichung	1994-2007	2 444 636	3 350	239.3 ± 90.5	19.9 ± 1.3	0.7 ± 0.8	9.7
	Kaohsiung	1994-2007	2 706 618	5 049	360.6 ± 133.6	30.1 ± 2.9	1.0 ± 1.1	13.2

[a]City-specific population size were averaged by using five-year census for Korea and Japan, and single-year for Taiwan, over the entire study period. Suicide and population data were obtained from Statistics Korea, Ministry of Strategy and Finance in South Korea, Ministry of Health, Labor and Welfare (for suicide) and Statistics Bureau, Ministry of Internal Affairs and Communications (for population) in Japan, and Department of Statistics, Ministry of Health and Welfare (for suicide) and Department of Statistics, Ministry of the Interior (for population) in Taiwan.

Table S2. Weather characteristics by season in fifteen cities.

Characteristic	Country	City	Spring (mean ± SD)	Summer (mean ± SD)	Autumn (mean ± SD)	Winter (mean ± SD)
Ambient temperature (°C)	Korea	Seoul	12.2 ± 6.0	24.4 ± 2.7	14.6 ± 6.7	-0.1 ± 4.4
		Busan	13.4 ± 4.5	23.6 ± 3.1	17.4 ± 5.3	5.0 ± 3.8
		Inchoen	11.2 ± 5.5	23.5 ± 2.8	14.8 ± 6.5	0.2 ± 4.1
		Daegu	14.0 ± 5.6	25.2 ± 3.2	15.8 ± 6.1	2.6 ± 3.5
		Daejeon	12.4 ± 5.9	24.4 ± 2.7	14.4 ± 6.4	0.5 ± 3.8
		Gwangju	13.0 ± 5.6	24.8 ± 2.7	15.8 ± 6.1	2.4 ± 3.6
	Japan	Sapporo	6.5 ± 5.9	19.8 ± 3.5	11.4 ± 6.3	-2.7 ± 3.4
		Sendai	10.0 ± 5.2	21.6 ± 3.7	15.0 ± 5.4	2.6 ± 3.0
		Tokyo	14.2 ± 5.0	25.0 ± 3.5	18.4 ± 5.1	6.9 ± 2.7
		Nagoya	13.8 ± 5.1	25.5 ± 3.1	17.9 ±5.6	5.4 ± 2.8
		Osaka	14.6 ± 5.1	26.5 ± 3.0	19.0 ± 5.4	6.9 ± 2.8
		Fukuoka	14.8 ± 4.6	26.0 ± 3.1	18.9 ± 5.1	7.5 ± 3.0
	Taiwan	Taipei	22.1 ± 4.1	28.9 ± 1.8	24.5 ± 3.2	17.2 ± 3.1
		Taichung	23.3 ± 3.7	28.3 ± 1.5	25.1 ± 2.7	17.9 ± 2.8
		Kaohsiung	25.4 ± 2.9	28.8 ± 1.4	26.4 ± 2.1	20.4 ± 2.5
Sunshine (hour)	Korea	Seoul	6.2 ± 4.0	4.6 ± 4.0	5.6 ± 3.4	5.2 ± 3.2
		Busan	6.6 ± 4.2	5.7 ± 4.4	6.2 ± 3.6	6.4 ± 3.2
		Inchoen	7.0 ± 4.1	5.7 ± 4.4	6.3 ± 3.5	5.9 ± 3.2
		Daegu	7.0 ± 4.1	5.2 ± 4.1	5.9 ± 3.5	6.3 ± 3.1
		Daejeon	6.9 ± 4.0	5.4 ± 4.1	5.9 ± 3.4	5.5 ± 3.1
		Gwangju	6.6 ± 4.1	5.0 ± 4.0	5.9 ± 3.4	5.3 ± 3.1
	Japan	Sapporo	6.5 ± 3.8	6.7 ± 4.3	5.2 ± 3.3	3.8 ± 2.5
		Sendai	7.3 ± 3.8	6.1 ± 4.0	5.7 ± 3.2	5.5 ± 2.6
		Tokyo	7.0 ± 3.8	6.1 ± 3.9	5.7 ± 3.3	6.7 ± 2.8
		Nagoya	7.7 ± 3.8	6.5 ± 3.9	6.4 ± 3.4	6.3 ± 2.8
		Osaka	7.0 ± 3.8	6.9 ± 3.8	6.0 ± 3.3	5.4 ± 2.7
		Fukuoka	7.0 ± 4.0	6.7 ± 4.0	6.1 ± 3.5	4.5 ± 3.0
	Taiwan	Taipei	3.3 ± 3.5	5.8 ± 3.6	4.3 ± 3.8	2.7 ± 3.2
		Taichung	5.1 ± 3.7	6.4 ± 3.7	6.5 ± 3.2	5.3 ± 3.3
		Kaohsiung	6.4 ± 3.5	6.5 ± 4.0	5.9 ± 3.0	5.6 ± 3.0
Relative humidity (%)	Korea	Seoul	57.5 ± 14.6	72.9 ± 11.7	63.8 ± 12.3	57.2 ± 13.5
		Busan	62.4 ± 16.8	80.2 ± 10.2	63.8 ± 14.3	47.9 ± 15.8
		Inchoen	65.2 ± 14.2	78.4 ± 10.3	67.2 ± 12.6	60.5 ± 13.5
		Daegu	52.7 ± 16.0	69.3 ± 11.3	63.7 ± 12.7	51.8 ± 14.2
		Daejeon	58.7 ± 14.3	74.8 ± 10.6	70.9 ± 10.3	64.0 ± 12.4
		Gwangju	61.5 ± 13.8	75.6 ± 9.6	68.4 ± 10.5	64.9 ± 12.2
	Japan	Sapporo	65.1 ± 12.5	75.0 ± 8.7	68.9 ± 9.9	69.8 ± 9.2
		Sendai	65.3 ± 14.8	81.4 ± 9.8	72.5 ± 11.6	65.2 ± 9.8
		Tokyo	60.2 ± 15.4	72.4 ± 9.0	65.5 ± 13.2	50.5 ± 14.2
		Nagoya	61.7 ± 14.6	72.4 ± 10.7	68.8 ± 11.4	63.7 ± 11.0
		Osaka	60.1 ± 12.4	68.1 ± 9.1	65.4 ± 9.8	61.0 ± 9.9
		Fukuoka	66.0 ± 13.1	73.9 ± 9.2	69.2 ± 9.9	63.4 ± 10.9
	Taiwan	Taipei	77.4 ± 9.4	74.1 ± 8.0	75.3 ± 9.0	77.7 ± 9.5
		Taichung	75.8 ± 7.8	76.5 ± 6.9	72.7 ± 7.0	74.3 ± 8.2
		Kaohsiung	75.2 ± 6.2	80.2 ± 6.6	75.6 ± 6.4	72.9 ± 7.1

Characteristic	Country	City	Spring (mean ± SD)	Summer (mean ± SD)	Autumn (mean ± SD)	Winter (mean ± SD)
Atmospheric pressure (hPa)	Korea	Seoul	1014.9 ± 6.2	1007.5 ± 4.1	1018.4 ± 5.9	1024.1 ± 5.2
		Busan	1014.8 ± 5.9	1008.2 ± 4.1	1017.4 ± 5.6	1021.9 ± 4.9
		Inchoen	1014.8 ± 6.2	1007.2 ± 4.2	1018.0 ± 5.8	1023.7 ± 5.2
		Daegu	1015.2 ± 6.2	1008.4 ± 4.2	1018.7 ± 5.9	1023.5 ± 5.2
		Daejeon	1015.2 ± 6.2	1007.6 ± 4.1	1018.5 ± 5.9	1024.2 ± 5.2
		Gwangju	1015.3 ± 6.0	1007.5 ± 4.1	1018.1 ± 5.8	1023.9 ± 4.9
	Japan	Sapporo	1012.3 ± 7.2	1009.3 ± 4.8	1014.8 ± 7.1	1013.6 ± 7.7
		Sendai	1014.3 ± 6.9	1010.0 ± 4.6	1016.4 ± 6.5	1015.9 ± 7.1
		Tokyo	1014.0 ± 6.8	1009.4 ± 4.6	1015.9 ± 6.4	1016.1 ± 6.9
		Nagoya	1014.7 ± 6.0	1009.3 ± 4.3	1016.1 ± 5.9	1018.6 ± 5.8
		Osaka	1015.0 ± 5.9	1008.9 ± 4.1	1016.5 ± 5.8	1019.7 ± 5.6
		Fukuoka	1015.2 ± 5.6	1008.2 ± 4.1	1016.8 ± 5.6	1021.6 ± 4.8
	Taiwan	Taipei	1012.3 ± 4.6	1005.2 ± 3.9	1013.0 ± 5.2	1019.5 ± 4.2
		Taichung	1002.7 ± 3.8	997.0 ± 3.9	1002.6 ± 4.4	1008.3 ± 3.2
		Kaohsiung	1012.1 ± 3.7	1006.6 ± 3.7	1011.8 ± 4.3	1017.6 ± 3.3

Season was defined by spring (March–May), summer (June–August), autumn (September–November), and winter (December–February). Study period varied depending on the country: Korea (1992–2010), Japan (1972–2010), and Taiwan (1994–2007). Weather data were obtained from the Korea Meteorological Administration, Japan Meteorological Agency, and Taiwan Central Weather Bureau.

Table S3. The associations for temperature or sunshine duration.

Country	City	ΔT (°C)[a]	PC[b] of Temperature without Sunshine (95% CI)	ΔS (hour)[c]	PC[b] of Sunshine with Temperature (95% CI)	PC[b] of Sunshine without Temperature (95% CI)
Korea	Seoul	5.1	7.3 (5.2, 9.4)	1.9	0.5 (-0.3, 1.4)	0.8 (-0.1, 1.7)
	Busan	4.0	7.4 (4.5, 10.5)	2.0	-0.2 (-1.4, 1.1)	0.8 (-0.4, 2.0)
	Inchoen	4.9	6.1 (2.2, 10.2)	1.9	-0.1 (-1.4, 1.3)	0.1 (-1.3, 1.5)
	Daegu	4.7	5.0 (1.1, 9.1)	1.9	-1.0 (-2.7, 0.8)	-0.4 (-2.1, 1.3)
	Daejeon	4.9	9.8 (4.2, 15.7)	1.9	1.9 (-0.4, 4.2)	2.5 (0.2, 4.8)
	Gwangju	4.6	5.4 (-0.5, 11.6)	1.9	1.7 (-0.6, 4.2)	2.0 (-0.3, 4.4)
Japan	Sapporo	4.8	3.4 (0.1, 6.8)	1.9	-0.3 (-1.6, 1.1)	-0.1 (-1.4, 1.3)
	Sendai	4.1	6.4 (1.8, 11.3)	1.8	-0.1 (-2.0, 1.9)	0.3 (-1.6, 2.2)
	Tokyo	3.9	5.2 (3.8, 6.6)	1.8	0.5 (-0.2, 1.1)	1.0 (0.4, 1.6)
	Nagoya	4.2	2.4 (-0.6, 5.5)	1.8	0.2 (-1.0, 1.5)	0.4 (-0.8, 1.6)
	Osaka	4.1	5.4 (3.0, 7.9)	1.7	0.1 (-0.9, 1.0)	0.6 (-0.4, 1.5)
	Fukuoka	3.9	4.3 (0.6, 8.2)	1.9	0.2 (-1.2, 1.7)	0.5 (-0.9, 1.9)
Taiwan	Taipei	2.6	6.5 (3.6, 9.5)	1.9	-0.5 (-2.1, 1.2)	1.1 (-0.4, 2.6)
	Taichung	2.4	8.1 (3.4, 12.9)	1.8	-0.7 (-3.2, 1.9)	1.1 (-1.2, 3.6)
	Kaohsiung	1.9	8.3 (4.7, 11.9)	1.7	-0.6 (-2.6, 1.4)	1.7 (0.0, 3.5)

[a]Percent change indicates suicide risks corresponding to a ΔT (or ΔS)-increase adjusting for relative humidity, atmospheric pressure, long-term time-trend, and month, with or without adjustment of sunshine duration (or temperature). [b]ΔT indicates a SD/2 of mean temperature, equal to standard deviation divided by two in mean temperature in each city. Similarly, [c]ΔS indicates a SD/2 of sunshine duration, equal to standard deviation divided by two in sunshine duration in each city.

Table S4. City-specific associations between same-day temperature and suicide according to gender and age group.

Characteristic	Category	Korea			Japan			Taiwan		
		city	estimate[a]	*p-value*[b]	city	estimate[a]	*p-value*[b]	city	estimate[a]	*p-value*[b]
Gender	Male	K01	7.9 (5.5, 10.3)	ref.	J01	3.8 (-0.3, 8.0)	ref.	T01	6.2 (2.6, 9.9)	ref.
	Female		5.9 (2.9, 9.0)	0.26		3.2 (-2.2, 8.8)	0.85		8.6 (4.0, 13.3)	0.31
Age	10-24 years		3.5 (-1.3, 8.5)	**0.01**		1.7 (-6.8, 11.0)	0.44		5.0 (-3.1, 13.8)	0.66
	25-64 years		6.5 (4.2, 8.9)	**0.01**		3.3 (-0.6, 7.4)	0.49		7.1 (3.5, 10.8)	0.99
	65≤		11.7 (7.8, 15.9)	ref.		6.0 (-1.1, 13.6)	ref.		7.1 (1.8, 12.7)	ref.
Gender	Male	K02	7.5 (4.0, 11.1)	ref.	J04	7.4 (1.4, 13.8)	ref.	T02	10.2 (4.4, 16.2)	ref.
	Female		7.7 (3.2, 12.5)	0.92		4.3 (-3.5, 12.7)	0.49		5.3 (-1.9, 13.1)	0.25
Age	10-24 years		11.6 (3.5, 20.3)	0.81		5.9 (-5.8, 19.1)	0.58		4.6 (-8.7, 19.9)	0.33
	25-64 years		5.7 (2.4, 9.2)	**0.02**		5.6 (-0.3, 11.8)	0.40		7.9 (2.3, 13.7)	0.32
	65≤		12.8 (7.0, 18.8)	ref.		10.4 (0.0, 21.8)	ref.		12.9 (3.6, 23.0)	ref.
Gender	Male	K03	6.7 (2.3, 11.3)	ref.	J13	5.0 (3.0, 7.0)	ref.	T03	9.3 (4.7, 14.1)	ref.
	Female		4.9 (-0.7, 10.9)	0.59		4.0 (1.6, 6.5)	0.46		8.2 (2.2, 14.5)	0.73
Age	10-24 years		3.4 (-6.0, 13.7)	0.39		4.5 (0.4, 8.7)	0.27		6.1 (-0.5, 18.5)	0.91
	25-64 years		5.8 (1.4, 10.3)	0.48		3.9 (2.0, 5.9)	**0.04**		9.9 (5.3, 14.7)	0.42
	65≤		8.4 (1.7, 15.5)	ref.		7.2 (4.2, 10.3)	ref.		6.9 (0.1, 14.1)	ref.
Gender	Male	K04	6.4 (1.8, 11.1)	ref.	J23	3.6 (-0.2, 7.6)	ref.			
	Female		3.8 (-1.9, 9.8)	0.43		1.1 (-3.5, 5.9)	0.34			
Age	10-24 years		6.1 (-3.4, 16.5)	0.57		4.1 (-4.2, 13.1)	0.67			
	25-64 years		4.4 (0.0, 9.0)	0.20		1.3 (-2.4, 5.1)	0.10			
	65≤		9.5 (2.0, 17.6)	ref.		6.3 (0.6, 12.3)	ref.			
Gender	Male	K05	9.8 (3.5, 16.6)	ref.	J27	5.2 (2.2, 8.4)	ref.			
	Female		7.6 (-0.2, 16.1)	0.63		5.0 (1.2, 9.0)	0.92			
Age	10-24 years		15.7 (2.1, 31.0)	0.69		6.4 (-0.6, 13.9)	0.68			
	25-64 years		7.1 (1.0, 13.6)	0.33		5.2 (2.2, 8.3)	0.85			
	65≤		12.3 (2.5, 23.1)	ref.		4.7 (0.2, 9.4)	ref.			
Gender	Male	K06	7.0 (0.4, 14.0)	ref.	J40	2.1 (-2.5, 6.9)	ref.			
	Female		0.4 (-7.5, 9.0)	0.15		9.2 (2.9, 16.0)	**0.04**			
Age	10-24 years		12.0 (-0.8, 26.4)	0.97		13.2 (2.2, 25.4)	0.25			
	25-64 years		2.1 (-4.1, 8.7)	0.13		3.0 (-1.5, 7.7)	0.56			
	65≤		11.6 (-0.2, 24.8)	ref.		5.4 (-2.3, 13.8)	ref.			

[a]A percent change of suicide risk corresponding to a SD/2-increase of mean temperature. [b]*p*-value for the difference in the association between suicide and temperature between men and women, or in those age 10–24 or 25–64 year compared with ≥65 years.

The abbreviations of cities stand for Seoul (K01), Busan (K02), Inchoen (K03), Daegu (K04), Daejeon (K05), Gwangju (K06), Sapporo (J01), Sendai (J04), Tokyo (J13), Nagoya (J23), Osaka (J27), Fukuoka (J40), Taipei (T01), Taichung (T02), and Kaohsiung (T03).

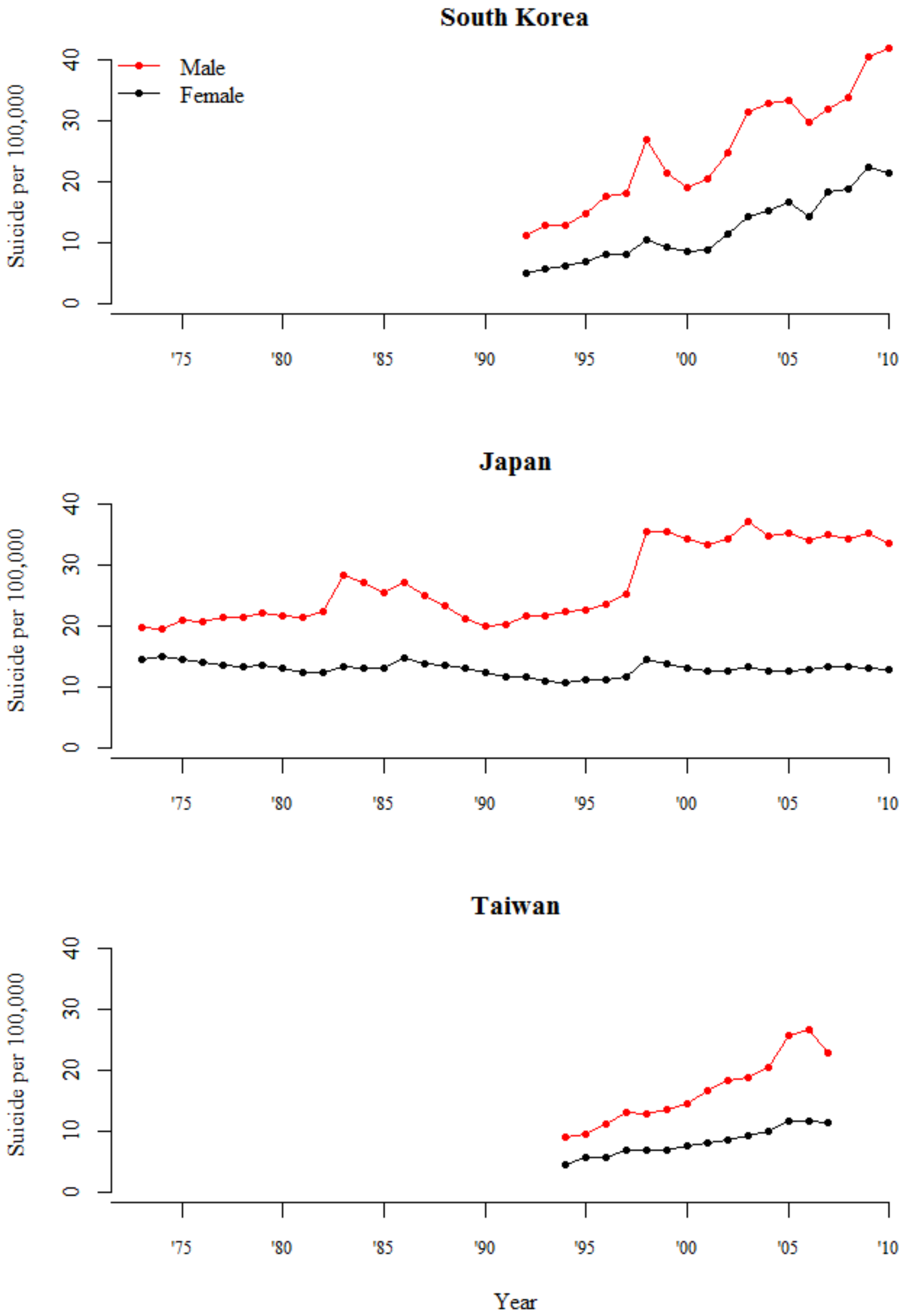

Figure S1. Yearly trend of suicide rates on national levels by gender in Korea, Japan, and Taiwan. Suicide and population data were obtained from Statistics Korea, Ministry of Strategy and Finance in South Korea, Ministry of Health, Labor and Welfare (for suicide) and Statistics Bureau, Ministry of Internal Affairs and Communications (for population) in Japan, and Department of Statistics, Ministry of Health and Welfare (for suicide) and Department of Statistics, Ministry of the Interior (for population) in Taiwan.

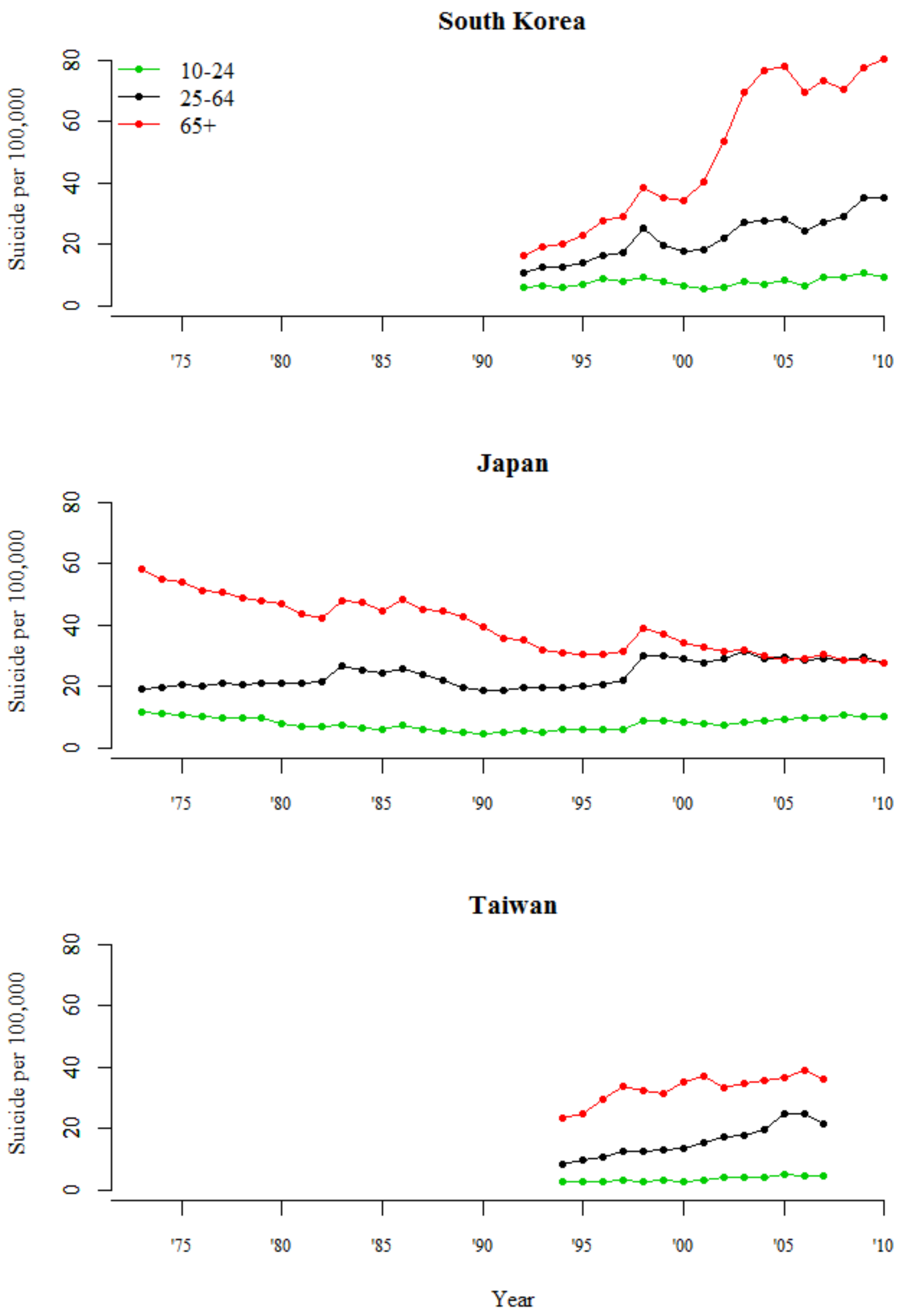

Figure S2. Yearly trend of suicide rates on national levels by age groups in Korea, Japan, and Taiwan. Suicide and population data were obtained from Statistics Korea, Ministry of Strategy and Finance in South Korea, Ministry of Health, Labor and Welfare (for suicide) and Statistics Bureau, Ministry of Internal Affairs and Communications (for population) in Japan, and Department of Statistics, Ministry of Health and Welfare (for suicide) and Department of Statistics, Ministry of the Interior (for population) in Taiwan.

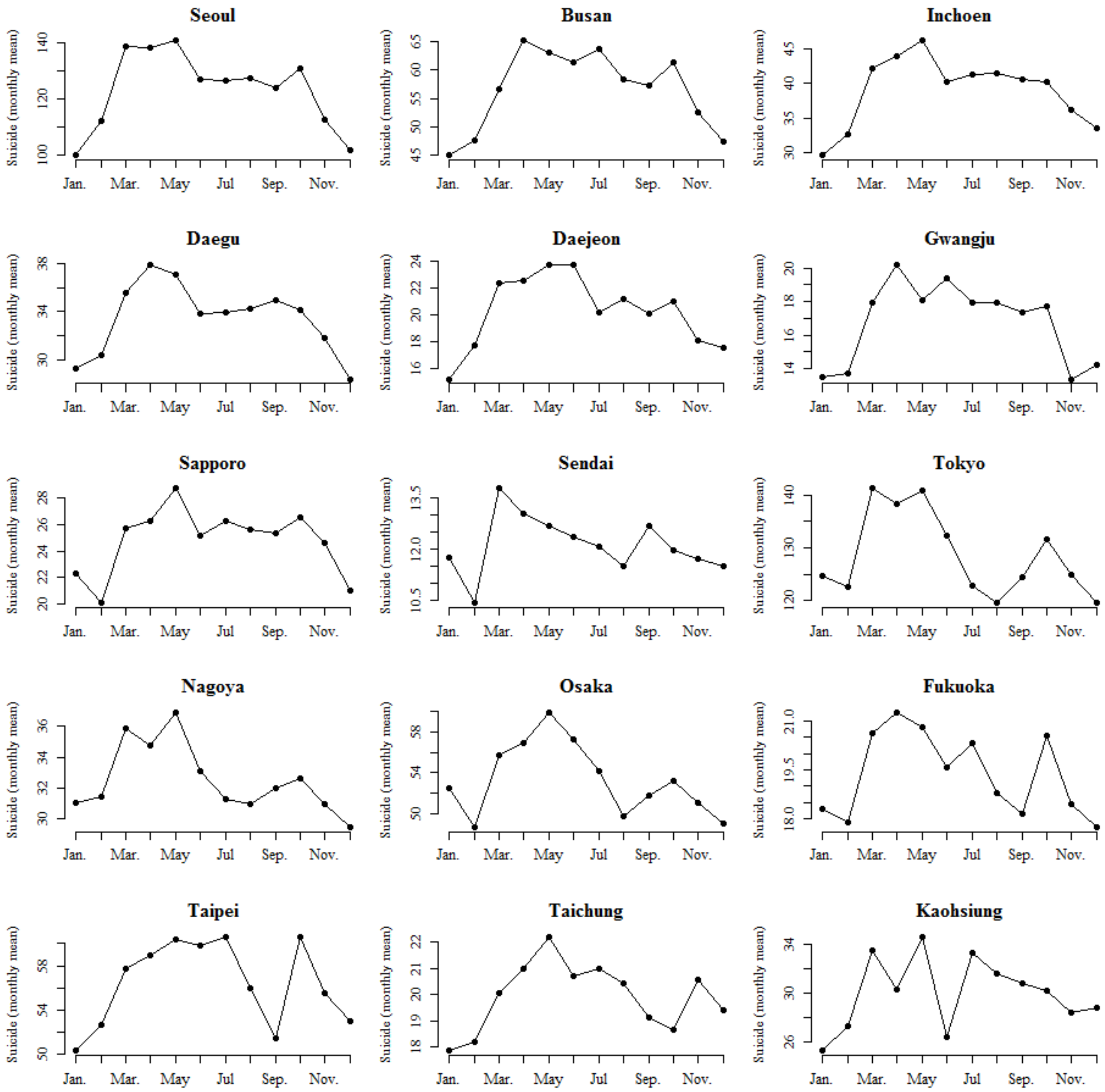

Figure S3. Monthly trend of suicide in fifteen cities. Study period varied depending on the country: Korea (1992–2010), Japan (1972–2010), and Taiwan (1994–2007). Suicide data were obtained from Statistics Korea, Ministry of Strategy and Finance in South Korea, Ministry of Health, Labor and Welfare in Japan, and Department of Statistics, Ministry of Health and Welfare in Taiwan.

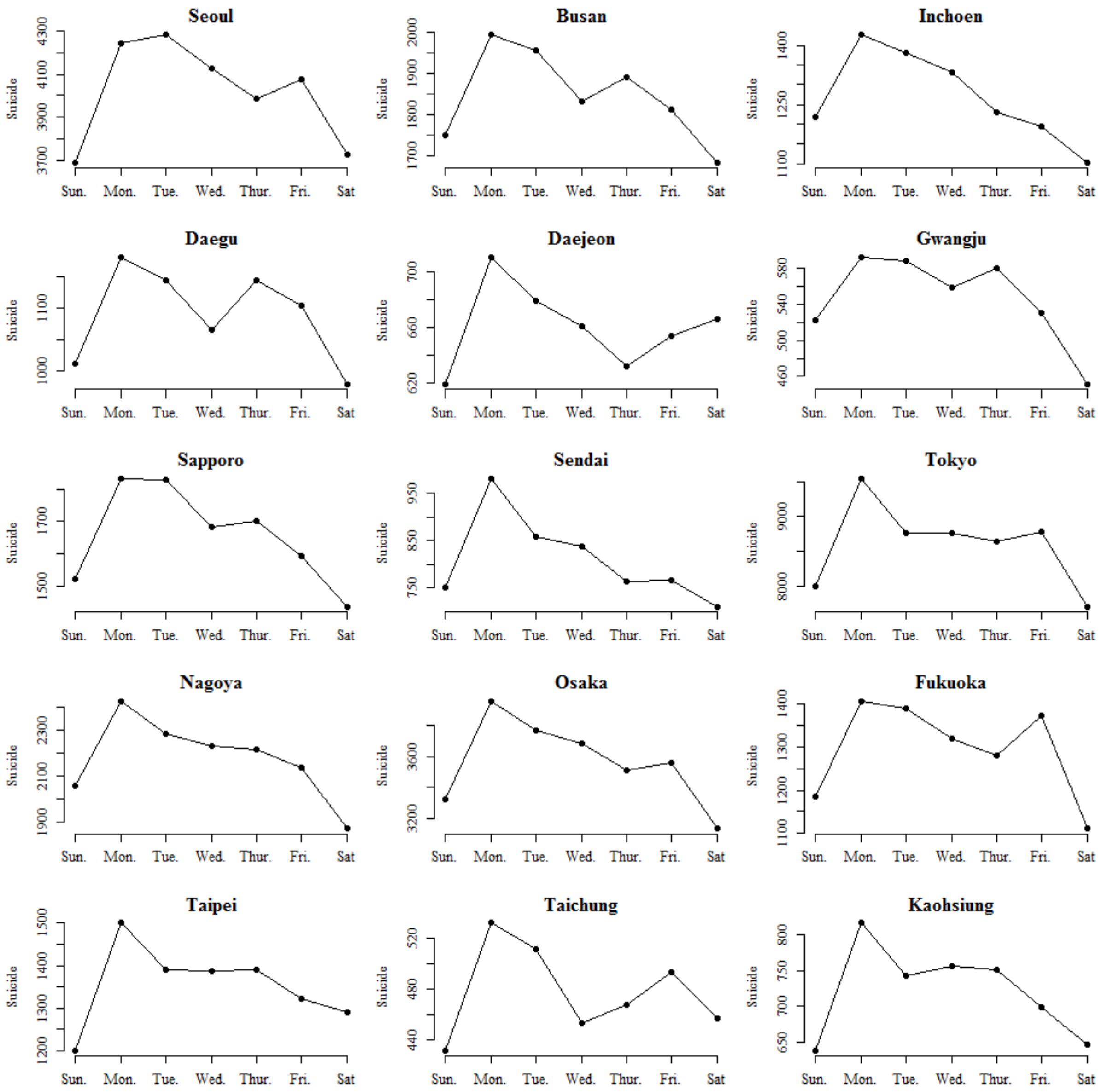

Figure S4. Trends for day of the week of suicide in fifteen cities. Study period varied depending on the country: Korea (1992–2010), Japan (1972–2010), and Taiwan (1994–2007). Suicide data were obtained from Statistics Korea, Ministry of Strategy and Finance in South Korea, Ministry of Health, Labor and Welfare in Japan, and Department of Statistics, Ministry of Health and Welfare in Taiwan.

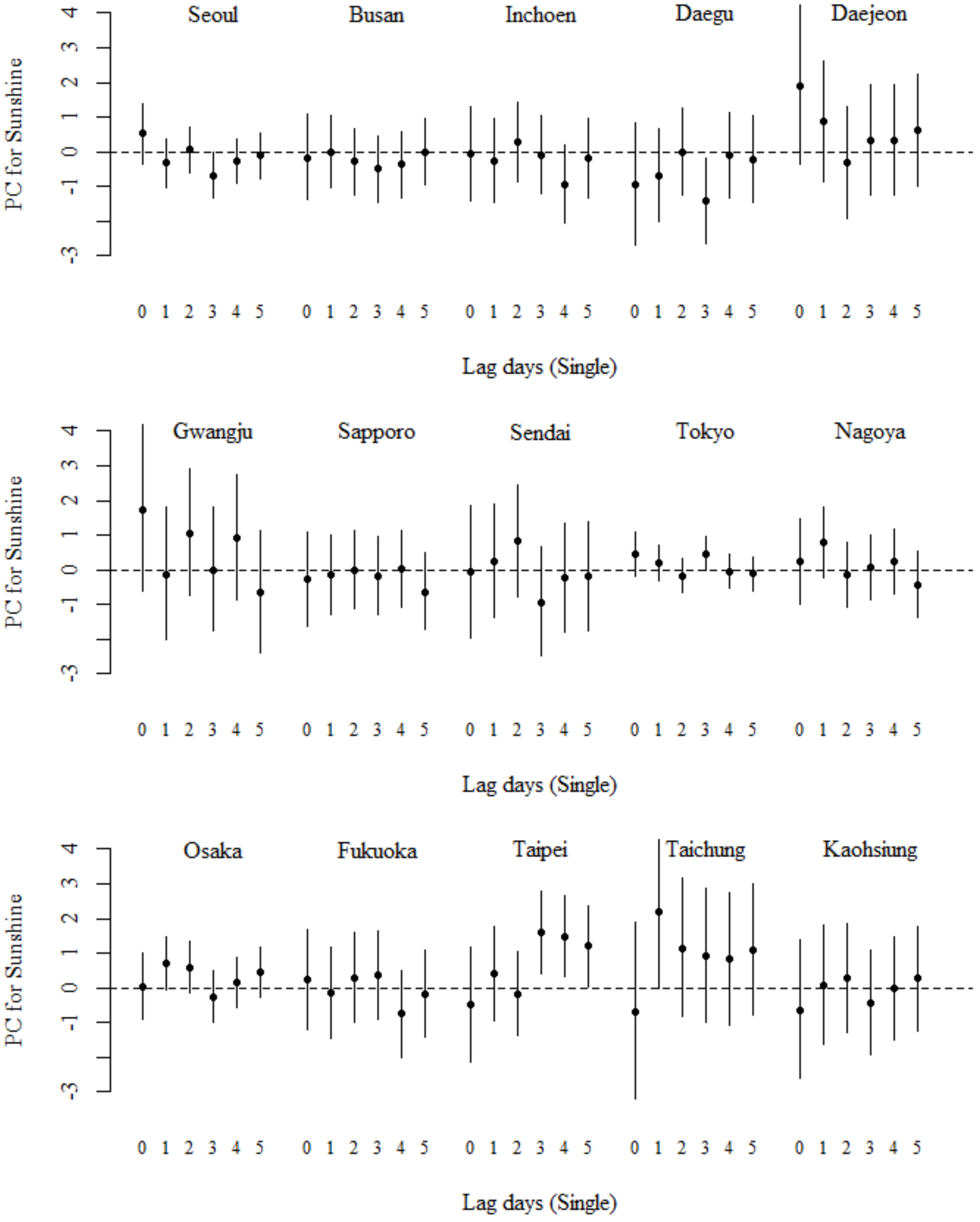

Figure S5. Lagged effect estimates for sunshine on suicide according to single lags. PC indicates percent change of suicide risk corresponding to a SD/2-increase of mean sunshine, adjusting for temperature, relative humidity, atmospheric pressure, long-term time-trend, and month.

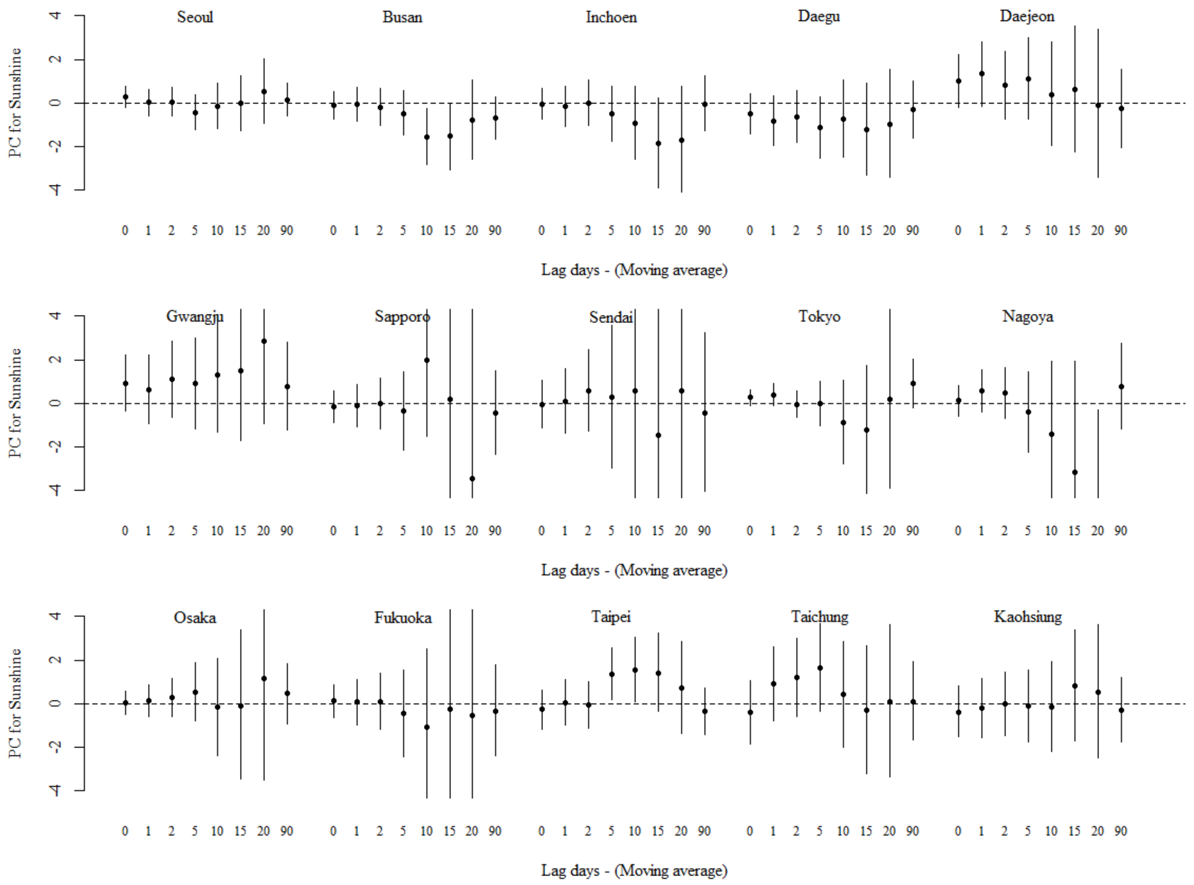

Figure S6. Lagged effect estimates for sunshine on suicide according to moving averages. Moving average 90 indicates the average of sunshine for a week on previous ninety days. PC indicates percent change of suicide risk corresponding to a SD/2-increase of mean sunshine, adjusting for temperature, relative humidity, atmospheric pressure, long-term time-trend, and month.

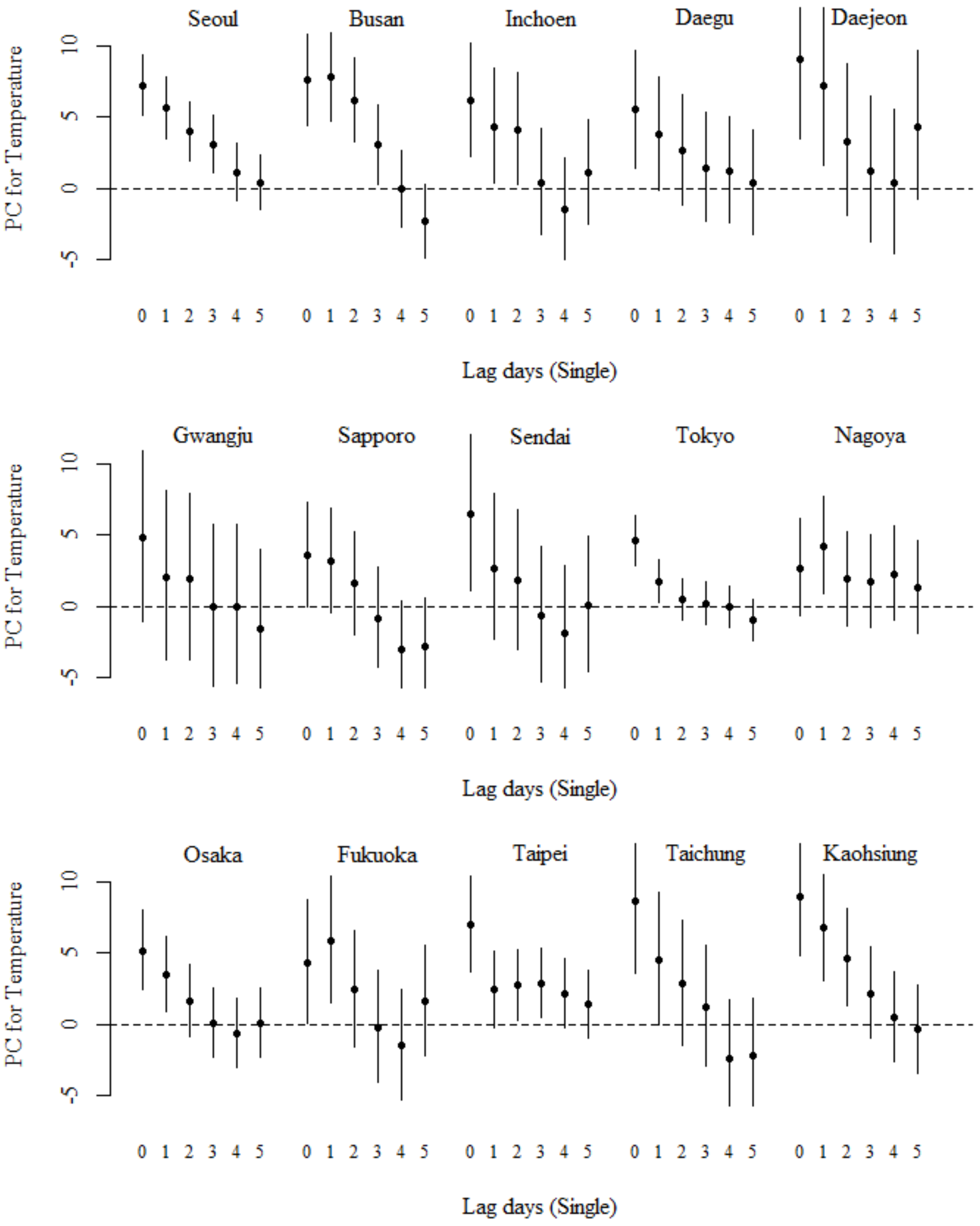

Figure S7. Lagged effect estimates for temperature on suicide according to single lags. PC indicates percent change of suicide risk corresponding to a SD/2-increase of mean temperature, adjusting for sunshine duration, relative humidity, atmospheric pressure, long-term time-trend, and month.

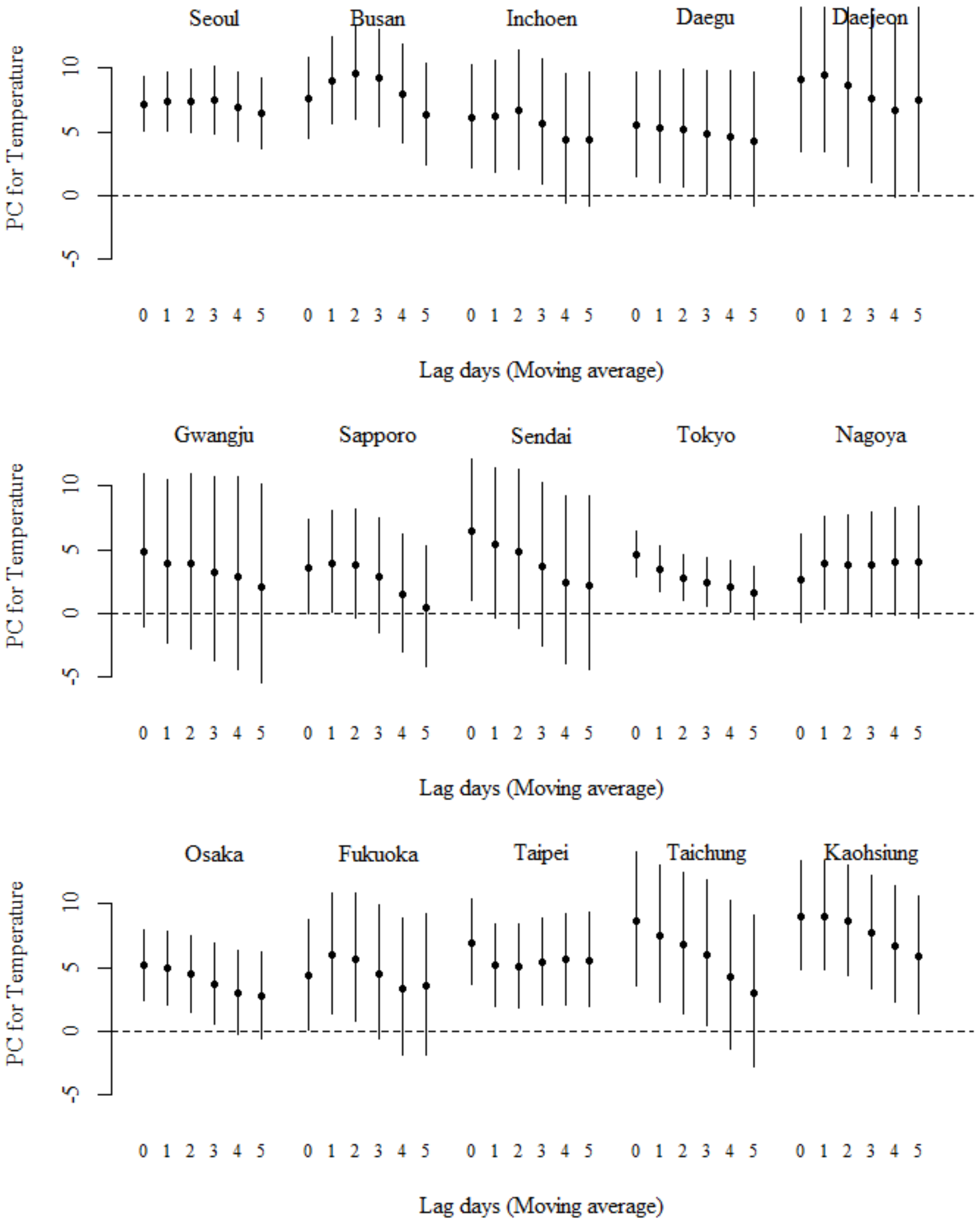

Figure S8. Lagged effect estimates for temperature on suicide according to moving averages. PC indicates percent change of suicide risk corresponding to a SD/2-increase of mean temperature, adjusting for sunshine duration, relative humidity, atmospheric pressure, long-term time-trend, and month.
